# Immune-metabolic interactions shape the fibrotic landscape of diabetic kidney disease: emerging mechanisms and therapeutic prospects

**DOI:** 10.3389/fphys.2025.1736472

**Published:** 2026-01-21

**Authors:** Yachan Gao, Xinxin Pang, Huichao Zhang, Dongdong Li, Jiarui Han, Zhenyi Chen, Xiaoyong Chen, Dongyang Li

**Affiliations:** 1 Henan Province Hospital of Traditional Chinese Medicine, The Second Affiliated Hospital of Henan University of Chinese Medicine, Zhengzhou, Henan, China; 2 The Second Clinical Medical College of Henan University of Chinese Medicine, Zhengzhou, Henan, China

**Keywords:** diabetic nephropathy, kidney fibrotic remodeling, metabo-immunomodulatory therapy, metabolic–immune interplay, mitochondrial metabolic remodeling

## Abstract

Diabetic kidney disease (DKD) is the leading cause of end-stage renal disease (ESRD), yet its progressive fibrosis cannot be solely attributed to hyperglycemia-induced oxidative stress or glomerular hypertension. Increasing evidence highlights that the bidirectional interaction between metabolic disturbances and immune activation—termed immunometabolic interactions—plays a pivotal role in driving DKD progression. Chronic metabolic stress, encompassing hyperglycemia, lipotoxicity, mitochondrial dysfunction, and gut-derived metabolites, reprograms innate and adaptive immune cells into pro-inflammatory and pro-fibrotic states. In turn, these activated immune cells exacerbate metabolic damage by promoting reactive oxygen species (ROS) overproduction, disrupting mitochondrial homeostasis, and facilitating extracellular matrix accumulation, thereby creating a self-amplifying loop that accelerates renal fibrosis. Key immunometabolic regulators, including HIF-1α, AMPK, mTOR, and SIRT1, coordinate metabolic signals with immune responses, providing novel mechanistic insights into DKD beyond traditional models. Recent therapeutic advances—such as Sodium-Glucose Cotransporter 2(SGLT2) inhibitors, GLP-1 receptor agonists, mineralocorticoid receptor antagonists, and multi-target natural compounds—offer renoprotective effects, partly by modulating these immunometabolic pathways. Fibrotic remodeling represents a core pathological tissue restructuring event in the kidney, typified by excessive extracellular matrix accumulation and irreversible structural destruction, which is coordinately propelled by the dual drivers of systemic metabolic disorders and local immune activation. A more precise characterization of immunometabolic alterations across disease stages, aided by single-cell and spatial multi-omics technologies, will be essential for identifying causal mechanisms rather than mere associations. Such discoveries could facilitate stage-specific, metabolism-immune–targeted interventions to prevent or slow fibrotic remodeling in DKD.

## 1 Introduction

Diabetic kidney disease (DKD) is a significant long-term complication affecting individuals with diabetes globally, and it is the primary cause of end-stage renal disease (ESRD) (Thomas et al., 2016; Alicic et al., 2017). Approximately one-third to two-fifths of people with diabetes will develop DKD. Chronic kidney failure substantially increases mortality risk, placing a considerable burden on healthcare systems worldwide (Saran et al., 2020; Jha et al., 2013; Reutens, 2013). Current treatments for chronic kidney disease (CKD) include angiotensin-converting enzyme inhibitors (ACEIs), angiotensin receptor blockers (ARBs), and strict blood pressure management. Sodium bicarbonate is also used to correct metabolic acidosis. However, these treatments are insufficient to halt disease progression, and the prognosis for patients remains poor (Pethő et al., 2024). Clinically, DKD is diagnosed through urine albumin and estimated Glomerular Filtration Rate(eGFR) tests, but these methods lack sensitivity in detecting early kidney scarring (de Boer et al., 2011). Therefore, understanding the key mechanisms underlying the onset and progression of DKD is critical for enhancing early diagnosis and developing targeted therapeutic strategies.

Renal fibrosis, marked by progressive glomerulosclerosis and tubulointerstitial fibrosis, is the final common pathological pathway of DKD (Duffield, 2014). Traditional models attribute fibrosis to metabolic disturbances (hyperglycemia, lipotoxicity), hemodynamic changes (glomerular hyperfiltration, hypertension), and chronic inflammation (Grabias and Konstantopoulos, 2014; Tuttle and Bruton, 1992). However, these classical models fail to fully capture the complexity and heterogeneity of DKD. Recent research emphasizes immune-metabolic interactions—the dynamic interplay between metabolic stress and immune activation—as a central driver of DKD progression (Narongkiatikhun et al., 2024).

Metabolic factors such as persistent hyperglycemia, accumulation of advanced glycation end products (AGEs), elevated free fatty acids (FFAs), and altered gut metabolites directly influence immune cell function. These cells subsequently secrete proinflammatory and profibrotic mediators, including tumor necrosis factor-α(TNF-α), interleukin-1β(IL-1β), and transforming growth factor-β1 (TGF-β1), thereby triggering inflammation and fibrosis and accelerating renal injury (Peng et al., 2024). Concurrently, mitochondrial dysfunction connects metabolic dysregulation to innate immune activation by impairing Adenosine Triphosphate (ATP) production and increasing reactive oxygen species (ROS) generation, thus promoting inflammasome activation and sustained renal inflammation (Liu et al., 2021).

Given these findings, a comprehensive mechanistic framework encompassing microvascular injury, metabolic stress, mitochondrial dysfunction, gut-kidney axis disruption, and dysregulated immune responses is essential for understanding DKD fibrosis. This review, therefore, focuses on the immune-metabolic network driving renal fibrogenesis in DKD. Specifically, this review discusses: microvascular hypoxia and endothelial-immune-metabolic coupling; metabolic reprogramming and immune activation; mitochondrial dysfunction as a metabolic-inflammatory nexus; gut-kidney axis disruption and bile acid-microbiota signaling in metabolic inflammation; and the therapeutic implications of targeting immune-metabolic-microbiota pathways.

## 2 Pathophysiological mechanisms and staged progression of renal fibrosis in DKD

DKD is a primary cause of ESRD, with renal fibrosis as the central pathological feature. Fibrosis progressively damages the structure and function of the glomeruli, tubulointerstitium, and blood vessels in the kidney, leading to irreversible scarring and kidney failure as the disease advances. Key histological changes include thickening of the glomerular basement membrane (GBM), expansion of the mesangial matrix, tubular atrophy, and excessive accumulation of extracellular matrix (ECM) (Efiong et al., 2025; Huang et al., 2023; Zhang et al., 2021; Humphreys, 2018). Glomerular injury is one of the earliest and most characteristic changes in DKD. Prolonged hyperglycemia and metabolic stress lead to GBM thickening and mesangial matrix expansion, marking the onset of fibrosis. The GBM, located between glomerular endothelial cells and podocytes, serves both structural and filtration roles (Marshall, 2016). A hyperglycemic microenvironment is a major inducer of progressive GBM thickening, with molecular mechanisms involving ECM synthesis, impaired ECM degradation, and aberrant glycosylation modifications (Wu et al., 2023).

Hyperglycemia activates the PI3K/Akt/mTOR signaling pathway, thereby promoting mesangial cell proliferation and upregulating the gene expression of collagen Ⅰ, collagen Ⅲ, and fibronectin, which ultimately leads to their excessive production and accumulation; High glucose levels and AGEs activate mesangial cells, resulting in the overproduction of collagen and fibronectin. Tubulointerstitial fibrosis is characterized by abnormal ECM accumulation. Fibroblasts and myofibroblasts are activated through epithelial-to-mesenchymal transition (EMT) and endothelial-to-mesenchymal transition (EndoMT), leading to the continuous production of type I and type III collagen. Microvascular rarefaction and hypoxia, due to the loss of peritubular capillaries (PTCs) and impaired blood flow, activate HIF-1α signaling, creating a vicious cycle of hypoxia and fibrosis. Metabolic and immune dysregulation further exacerbates the condition, with disrupted immune activity and metabolic reprogramming driving the sustained release of inflammatory cytokines, chemokines, and profibrotic factors. These processes contribute to DKD progression and fibrotic remodeling.

Glomerular EphrinB2/Epac1-Rap1 signaling mitigates proteinuria by regulating mitochondrial bioenergetics in DKD. Tubulointerstitial epithelial cells undergo EMT, transforming into fibroblasts, accompanied by distinct pathology and immunometabolic interactions. Hyperglycemia disrupts E-cadherin/β-catenin signaling, inducing β-catenin nuclear translocation, which activates fibrotic genes (Ni et al., 2025). Mitochondrial dysfunction and BNIP3-mediated mitophagy impairment exacerbate tubular apoptosis and inflammation. Inhibition of KCa3.1 restores mitochondrial quality and reduces fibrosis by 40% compared to controls (*p* < 0.05 (21)). PTC rarefaction is critical for interstitial fibrosis. Diabetic endothelial metabolic reprogramming impairs vascular endothelial growth factor (VEGF) signaling and reduces vascular density (Ni et al., 2025), while mesenchymal stem cells (MSCs) repair capillaries through paracrine effects, increasing capillary density by 30% and downregulating fibrotic markers (Ni et al., 2025). Intercellular crosstalk forms a local fibrosis microenvironment network, and targeted therapies aimed at these mechanisms may delay fibrosis progression (Ni et al., 2025; Xu et al., 2022).

**FIGURE 1:**
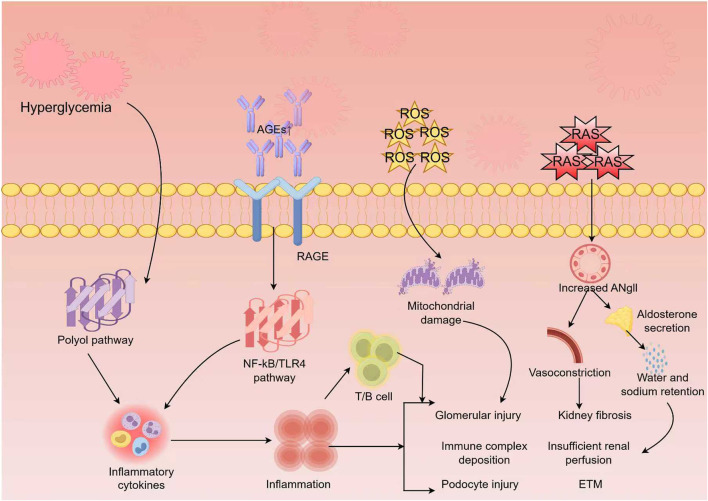
Stages of diabetic kidney disease (DKD) fibrosis. Persistent hyperglycemia and dyslipidemia drive DKD progression from inflammation to fibrosis. The inflammatory phase features immune activation and ECM deposition; the transition phase involves metabolic–immune interaction and myofibroblast activation; and the chronic fibrosis phase is characterized by ECM imbalance and irreversible renal scarring. By Figdraw.

Recent research has revealed that fibrosis in DKD is not a straightforward, linear process. Rather, DKD progresses through several distinct and dynamic stages. The early inflammatory phase is characterized by acute immune activation, triggered by persistent hyperglycemia, oxidative stress, and glomerular hyperfiltration, all of which cause damage to endothelial and tubular epithelial cells. These damaged cells release damage-associated molecular patterns (DAMPs), which activate the innate immune system. This leads to the recruitment of monocytes and macrophages to the kidney, where they polarize toward the pro-inflammatory M1 phenotype. These macrophages release tumor necrosis factor-α (TNF-α), interleukin-1β (IL-1β), and C–C motif chemokine ligand 2 (CCL2), which further damage the basement membrane and initiate the early accumulation of ECM components (Nikolic-Paterson et al., 2014; Wynn and Barron, 2010). As DKD progresses, a new phase emerges, marking a shift from short-term to long-term inflammation. This stage is characterized by a stronger interplay between immune responses and metabolic disturbances. At this point, macrophages transition into the M2 phenotype and begin secreting profibrotic factors such as TGF-β1 and Platelet-Derived Growth Factor (PDGF). These factors directly activate fibroblasts and myofibroblasts, accelerating fibrosis (Wynes et al., 2004; Floege et al., 2008). Concurrently, dysregulations in glucose and lipid metabolism, along with mitochondrial dysfunction, alter the energy metabolism of immune cells, leading them to rely more heavily on glycolysis, resulting in lactate accumulation. These metabolic byproducts act as signals that modulate immune cell behavior, thus establishing a problematic connection between metabolism and immunity (Narongkiatikhun et al., 2024; Darshi et al., 2024; Yang et al., 2017). In the final stage, ECM homeostasis is disrupted. The balance between ECM synthesis and degradation is lost, with significant disturbances in the equilibrium of matrix metalloproteinases (MMPs) and tissue inhibitors of metalloproteinases (TIMPs). This imbalance results in excessive ECM accumulation, leading to tubular atrophy, loss of microvasculature, and ultimately irreversible kidney scarring (Takamiya et al., 2013; Han et al., 2006). Notably, these stages are not completely distinct but overlap, exacerbating each other. Throughout the progression of DKD, renal fibrosis exhibits stage-specific clinical features and mechanisms. Early DKD is marked by glomerular hyperfiltration, microalbuminuria, and mild fibrosis, including GBM thickening and mesangial matrix expansion. In contrast, advanced stages show severe fibrosis and progressive renal dysfunction (Martinez Leon et al., 2026). EphrinB2 signaling plays a critical role in inhibiting early fibrosis by regulating Epac1-Rap1-mediated mitochondrial function. However, its downregulation in later stages promotes uncontrolled fibrosis. Immune cell infiltration and microRNA regulation shift from anti-inflammatory to pro-inflammatory (Xu et al., 2022), sustaining chronic inflammation throughout the disease. Meanwhile, metabolic disturbances exacerbate immune damage in the middle and late stages. In the final stage, excessive ECM production leads to permanent scarring. These findings emphasize the pivotal role of the immune-metabolic interaction in initiating and progressing renal fibrosis in DKD. Targeting key immunometabolic pathways early in the disease could significantly slow, or even reverse, the fibrotic process, offering a promising approach to more precise, stage-specific treatments (Figure 1).

## 3 Metabolism–immunity crosstalk in diabetic kidney disease fibrosis

**FIGURE 2:**
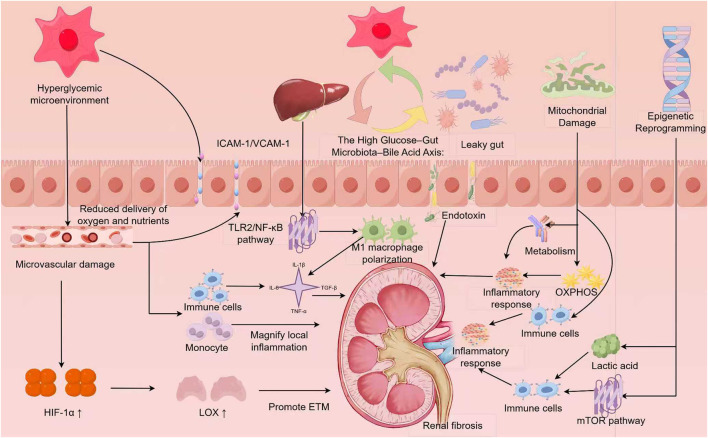
Metabolism–immunity crosstalk in diabetic kidney disease (DKD). Chronic metabolic stress in diabetes triggers microvascular injury, hypoxia, and metabolic reprogramming in renal and immune cells. These changes enhance immune cell infiltration and polarization (M1/M2 macrophages, Th17/Treg cells), while gut dysbiosis and bile acid imbalance amplify systemic inflammation. Mitochondrial dysfunction releases ROS and DAMPs, activating innate immune pathways and promoting fibrosis. Metabolic intermediates such as succinate, NAD^+^, and lactate further drive epigenetic remodeling, forming a persistent inflammatory and fibrotic state. By Figdraw.

In DKD, systemic metabolic disturbances and immune inflammation are intricately connected. Persistent hyperglycemia, dyslipidemia, and insulin resistance lead to the accumulation of glucose, FFAs, branched-chain amino acids (BCAAs), branched-chain keto acids (BCKAs), lactate, and succinate within cells. These molecules not only serve as energy substrates but also function as signaling molecules that trigger inflammation. Upon activation, particularly of M1 and M2 macrophages and Th17 cells, immune cells release cytokines such as TNF-α, IL-1β, and Interleukin-6(IL-6). These inflammatory mediators impair mitochondrial oxidative phosphorylation (OXPHOS) in renal tubular epithelial and endothelial cells. As oxidative metabolism diminishes, cells increasingly rely on glycolysis, resulting in inefficient ATP production, excessive ROS, and the accumulation of toxic metabolic intermediates. This creates a vicious cycle: metabolic dysfunction exacerbates immune activation, which in turn drives further metabolic damage. This feedback loop accelerates ECM deposition and fibrosis. Understanding this bidirectional relationship offers insight into the progression of DKD from early metabolic disturbances to late-stage irreversible fibrosis. Therapeutically, targeting immunometabolic checkpoints and their pathways presents a promising approach. Reprogramming immune cell metabolism to restore the balance between metabolic and inflammatory signals can reduce chronic inflammation and limit kidney fibrosis (Figure 2).

### 3.1 Microvascular injury and hypoxia: the pathological initiation of fibrosis

The development of fibrosis in DKD begins with injury to the small blood vessels in the kidney. Persistent hyperglycemia thickens the GBM, damages podocytes, and leads to the loss of PTCs. These changes reduce the delivery of oxygen and nutrients, creating conditions conducive to progressive tissue damage (Satchell and Tooke, 2008; Kida, 2020). As these small vessels are lost and blood flow decreases, a state of chronic hypoxia develops. In response, the HIF-1α signaling pathway is activated.

In the early stages of DKD, HIF-1α activation serves as an adaptive mechanism to protect kidney tissue. It regulates cellular energy metabolism and promotes angiogenesis. Under acute hypoxia, HIF-1α increases glucose uptake and upregulates glycolytic enzymes to maintain energy production in the absence of sufficient oxygen. Additionally, it upregulates target genes such as erythropoietin (EPO) and VEGF, improving local oxygen supply and supporting capillary regeneration (Nangaku et al., 2013). Short-term or moderate activation of HIF-1α can mitigate ischemic and early diabetic kidney injury, suggesting the presence of a “compensatory protective window” during the acute phase of DKD.

However, under chronic hyperglycemia and prolonged hypoxia, sustained HIF-1α activation becomes detrimental rather than protective. Continuous stabilization of HIF-1α in renal tubular epithelial cells increases the expression of extracellular matrix-modifying enzymes, such as lysyl oxidase (LOX), promoting EMT and enhancing cell motility, which drives tubulointerstitial fibrosis. In contrast, genetic deletion of HIF-1α in epithelial cells significantly reduces fibrosis in the unilateral ureteral obstruction (UUO) model, with decreased inflammatory cell infiltration and collagen deposition (Han et al., 2006). Moreover, chronic hypoxia not only perpetuates inflammation but also attracts profibrotic progenitor cells and impairs stem cell regenerative capacity (Fine and Norman, 2008). These combined effects accelerate interstitial scarring, leading to the gradual loss of kidney function (Martinez Leon et al., 2026). Thus, HIF-1α plays a dual role in DKD: it protects kidney tissue during acute injury but contributes to inflammation and fibrosis under long-term stress. This “two-phase effect” highlights the complex behavior of hypoxia signaling at different stages of DKD and suggests that adjusting HIF-1α activity at specific time points may offer therapeutic potential.

Microvascular injury and sustained hypoxia create the conditions for renal fibrosis in DKD, primarily through the continuous activation of HIF-1α and its downstream pathways that promote fibrosis (Figure 2; Table 1).

**TABLE 1:** Mechanistic summary of metabolism–immunity crosstalk driving renal fibrosis in diabetic kidney disease (DKD).

Interaction	Key pathways/molecules	Mechanism	Impact on DKD fibrosis	Refs
High glucose–metabolite–immune activation	Hyperglycemia, AGEs, glucose/FFA/BCAA/BCKA, lactate, succinate	Hyperglycemia and accumulated metabolites activate mesangial and immune cells, enhance inflammatory signaling, and suppress mitochondrial OXPHOS.	Establishes a metabolic–immune amplification loop that accelerates ECM deposition and early fibrotic remodeling	Narongkiatikhun et al. (2024), Wu et al. (2023), Wynn and Barron (2010), Wynes et al. (2004), Floege et al. (2008), Darshi et al. (2024), Yang et al. (2017), Takamiya et al. (2013)
Microvascular injury and hypoxia	GBM thickening, mesangial expansion, PTC rarefaction, HIF-1α, VEGF, LOX, EphrinB2/Epac1-Rap1, KCa3.1, MSCs	Structural microvascular damage leads to chronic hypoxia and sustained HIF-1α activation, driving EMT/EndoMT and fibroblast activation	Forms the hemodynamic and metabolic foundation for progressive tubulointerstitial fibrosis	Efiong et al. (2025), Huang et al. (2023), Zhang et al. (2021), Humphreys, 2018; Marshall (2016), Wu et al. (2023), Ni et al. (2025), Huang et al. (2022), Xu et al. (2022), Han et al. (2006), Martinez Leon et al. (2026), Satchell and Tooke (2008), Kida (2020), Nangaku et al. (2013), Higgins et al. (2007)
Immune cell metabolic reprogramming	ICAM-1/VCAM-1, M1/M2 glycolysis–OXPHOS shift, Th17 glycolysis, Treg FAO, SUCNR1, lactate–HIF-1α, tubular exosomes, RIPK1/RIPK3/MLKL, JAML/SIRT1	Adhesion molecules recruit immune cells; hypoxia and metabolites reprogram macrophages and T cells toward inflammatory/profibrotic states	Amplifies cytokine release, tubular injury, and interstitial ECM accumulation throughout disease progression	Hill et al. (1995), Siddiqui et al. (2023), Cervantes et al. (2024), Okada et al. (2003), Tang and Yiu (2020), Zh et al. (2025), Bonacina et al. (2019), Pei et al. (2025), Youssef et al. (2024), Pu et al. (2024), Ye et al. (2022), Jiang et al. (2024), Jia et al. (2022), Wang D. et al. (2024), Wang Y.et al. (2024) Qu et al. (2023), Smith et al. (2017), Chen L. et al. (2023) Liu et al. (2025), Ma T. et al. (2025)
High glucose–gut microbiota–bile acid axis	LPS–TLR4/MyD88–NF-κB, MAVS, FXR/TGR5, 12α-OH bile acids, 3-oxoLCA, isoalloLCA, ADMA, methylamines, uremic solutes	Hyperglycemia induces dysbiosis, endotoxemia, impaired bile acid signaling, and abnormal gut-derived metabolites	Sustains systemic and renal inflammation, enhances metabolic stress, and accelerates multiorgan fibrotic progression	Wada and Makino (2016), de Melo et al. (2022), Tao et al. (2019), Lassenius et al. (2011), Nymark et al. (2009), Wang M. et al. (2023) Li et al. (2024), Linh et al. (2022), Tian et al. (2023), Corradi et al. (2024), Schrauben et al. (2023), Saulnier et al. (2025), Sapa et al. (2022), Kuo et al. (2025), Tao et al. (2024), Shi et al. (2023), Stepanova (2024), Forbes and Thorburn (2018)
Mitochondrial damage	Impaired OXPHOS, mtROS, BNIP3-mitophagy deficiency, P53–Bax/Bcl-2, DAMPs (HMGN1), GLUT1, NETs, NLRP3, MAMs–Ca^2+^, TRPV1–AMPK–Fundc1	Hyperglycemia disrupts OXPHOS, increases mtROS and DAMP release, impairs mitophagy, and induces MAM-mediated Ca^2+^overload and inflammasome activation	Links mitochondrial dysfunction to sterile inflammation and fibrosis in tubular, glomerular, and immune compartments	Darshi et al. (2024) Zhao et al. (2024) Aachoui et al. (2013) Yu et al. (2024) Song et al. (2024) Wang M. et al. (2023) Ma Y. et al. (2025) Murphy and O’Neill (2020) Thorens and Mueckler (2010) Basso et al. (2021) Woodfin et al. (2016) Gupta et al. (2022) Shafqat et al. (2022), Zheng et al. (2022) Wei et al. (2020), Shahzad et al. (2022) Tannahill et al. (2013)
Epigenetic remodeling	Succinate, α-KG, NAD^+^/SIRT1, DNMT1–mTOR, PFKFB3, histone lactylation (H4K12la, H3K18la), m^6^A/METTL3, CTSS, CD53, ATP-dependent chromatin remodeling	Metabolic intermediates drive DNA/histone/RNA modifications (methylation, acetylation, lactylation, m^6^A), forming persistent inflammatory programs	Creates a “metabolic–inflammatory memory” that maintains profibrotic gene expression and promotes irreversible fibrosis	Wang W. et al. (2019) Chen et al. (2019), Wang J. et al. (2024) Shi et al. (2025), Chu et al. (2021), Wang H. et al. (2025) Jung et al. (2024), Leilei et al. (2025) Chen et al. (2024)
Immuno-metabolic signaling crosstalk	NF-κB, HIF-1α, TGF-β/Smad, JAK/STAT, PI3K/Akt, AMPK, mTOR, NLRP3	Inflammatory pathways integrate with metabolic sensors; metabolic stress chronically activates immune signaling	Forms a self-reinforcing vicious cycle that drives DKD from early immune activation to late-stage fibrosis	Wynn and Barron (2010) Wynes et al. (2004) Floege et al. (2008) Darshi et al. (2024) Han et al. (2006) Zh et al. (2025) Wada and Makino (2016) de Melo et al. (2022) Tao et al. (2019), Zhao et al. (2024) Shahzad et al. (2022) Tannahill et al. (2013) Wang W. et al. (2019) Chen et al. (2019) Wang Y. et al. (2024) Chu et al. (2021) Wang H. et al. (2025) Jung et al. (2024) Leilei et al. (2025) Chen et al. (2024)

### 3.2 Hypoxia-induced metabolic reprogramming: the energetic basis for immune amplification

A hypoxic microenvironment significantly disrupts cellular energy metabolism, driving metabolic reprogramming that sustains immune activation and inflammation. In conditions of chronic hyperglycemia and low oxygen, endothelial cells upregulate the expression of intercellular adhesion molecule-1 (ICAM-1) and vascular cell adhesion molecule-1 (VCAM-1) (Hickey and Martin, 2013; Hill et al., 1995). These adhesion molecules facilitate the attachment, migration, and infiltration of monocytes into renal tissue, amplifying local inflammatory responses (Siddiqui et al., 2023). In diabetic models, ICAM-1 deficiency or inhibition of ICAM-1/LFA-1 interactions significantly reduces macrophage accumulation and alleviates renal inflammation (Cervantes et al., 2024; Okada et al., 2003). These findings highlight adhesion molecule-mediated immune recruitment as a critical early event in DKD-associated immune activation.

Renal biopsy studies demonstrate that macrophages are the predominant infiltrating immune cells throughout all stages of DKD, from early to advanced disease. CD4^+^ T cells—particularly Th1 and Th17 subsets—and activated B cells also increase markedly. The abnormal presence and activity of these immune cells correlate closely with the severity of proteinuria and the progressive decline in renal function (Tang and Yiu, 2020; Zh et al., 2025; Bonacina et al., 2019).

Among these infiltrating immune cells, macrophages play a central role. M1 macrophages rely primarily on glycolysis for rapid ATP production, supporting cytokine secretion and inflammatory responses. HIF-1α activates hexokinase 2 (HK2) to promote glycolysis, inducing the secretion of IL-6, IL-1β, and TNF-α, and exacerbating glomerular hypertrophy and renal tubular injury (Pei et al., 2025). In contrast, M2 macrophages predominantly rely on OXPHOS and fatty acid oxidation. Under chronic stimulation, M2 macrophages shift their phenotype and begin releasing profibrotic mediators such as transforming growth factor-β1 (TGF-β1) and platelet-derived growth factor (PDGF), which promote tissue remodeling and fibrosis (Youssef et al., 2024). Metabolic intermediates also serve as key regulatory signals in this process. For example, succinate activates its receptor Succinate Receptor (SUCNR)1, driving M2 macrophage polarization and enhancing the expression of profibrotic molecules, including PDGF and connective tissue growth factor (CTGF) (Pu et al., 2024). Conversely, lactate stabilizes HIF-1α and drives macrophages toward a pro-inflammatory phenotype (Ye et al., 2022; Jiang et al., 2024). Additionally, exosomes derived from tubular epithelial cells can stabilize HIF-1α in macrophages, enhancing glycolytic flux and intensifying both inflammatory and fibrotic responses (Jia et al., 2022).

T cells are also intricately influenced by their metabolic state. Th17 cells primarily rely on glycolysis to support proliferation and the production of interleukin-17 (IL-17), a cytokine that drives tubular inflammation and stimulates ECM synthesis, thereby contributing to renal fibrosis (Wang D. et al., 2024). In contrast, regulatory T cells (Tregs) depend on fatty acid oxidation to maintain their immunosuppressive function. In DKD, metabolic disturbances in Tregs impair their function, resulting in an elevated Th17/Treg ratio and disrupting immune homeostasis, which perpetuates chronic inflammation (Wang J. et al., 2024). Other immune cells also play significant roles in DKD. Dendritic cells (DCs) mature under high-glucose conditions, releasing inflammatory cytokines and upregulating molecules that activate other immune cells (Qu et al., 2023). Additionally, B cells produce harmful antibodies and aberrant cytokines, exacerbating podocyte injury and increasing inflammation in renal tissue (Smith et al., 2017) (Table 1).

Podocyte injury and loss are critical events in the progression of DKD, driving renal fibrosis. Chronic microinflammation, induced by hyperglycemia, triggers necroptosis in podocytes, activating key proteins such as TNF-α and p-MLKL, and forming the RIPK1/RIPK3/MLKL signaling axis. This axis further amplifies inflammation and injury, promotes ECM deposition, and activates the TGF-β1/Smad signaling pathway, which plays a pivotal role in renal fibrosis (Chen J. X. et al., 2023). Additionally, the JAML/SIRT1 signaling pathway, central to renal lipid metabolism, is closely linked to podocyte function. Dysregulation of this pathway in DKD results in abnormal lipid accumulation in the kidney, exacerbating podocyte dysfunction and worsening renal damage (Liu et al., 2025).

In summary, hypoxia-induced immune metabolic reprogramming acts as a critical link between environmental stress and immune system overactivation. Activated immune cells release pro-inflammatory cytokines such asM1 macrophages, Th17 cells, IL-1β, TNF-α, and IL-6, as well as growth factors like TGF-β, along with other damaging substances. These signals maintain a highly inflammatory and profibrotic environment in the kidney, driving DKD toward progressive and irreversible fibrosis (Tang and Yiu, 2020). In renal tubular epithelial cells, this metabolic reprogramming disrupts their energy metabolism and alters communication with nearby immune cells. This modified communication enhances paracrine signaling, worsening local inflammation and accelerating disease progression (Figure 2).

### 3.3 The high glucose–gut microbiota–bile acid axis: systemic amplification and feedback of metabolism–immunity crosstalk

Persistent hyperglycemia remains the primary pathological driver of DKD. During prolonged hyperglycemia, glomerular and tubular epithelial cells produce increased amounts of AGEs and ROS. These substances bind to receptors for AGEs (RAGE), triggering NF-κB signaling activation. As a result, inflammatory mediators such as IL-1β, TNF-α, and monocyte chemoattractant protein-1 (MCP-1) are elevated, promoting the infiltration of monocytes and macrophages into kidney structures (Ma Y. et al., 2025). Simultaneously, high glucose conditions enhance Toll-like receptor (TLR) signaling, particularly through the TLR4-myeloid differentiation primary response 88 (MyD88)-NF-κB pathway, which further amplifies inflammation and exacerbates renal damage (Wada and Makino, 2016; de Melo et al., 2022) (Table 1).

Beyond local kidney effects, hyperglycemia and insulin resistance disrupt systemic lipid metabolism and trigger inflammation through immune regulation mediated by the gut microbiota. Patients with DKD frequently exhibit gut microbiota dysbiosis, characterized by decreased microbial diversity and an imbalance between beneficial and pathogenic bacteria (Tao et al., 2019). For instance, an increase in *Shigella* species compromises intestinal barrier integrity, resulting in an “intestinal leakage” phenotype. This damage allows lipopolysaccharide (LPS) from gut microbes to enter the bloodstream, inducing metabolic endotoxemia. Circulating LPS interacts with TLR4 on macrophages, triggering the release of IL-6 and IL-1β, thus maintaining a state of chronic low-grade inflammation (Lassenius et al., 2011) (Figure 2). Both clinical and experimental studies consistently show a positive correlation between serum LPS levels, urinary albumin excretion, and the severity of renal fibrosis (Nymark et al., 2009; Wang M. et al., 2023). These findings suggest that enterogenic endotoxemia directly contributes to kidney damage and fibrosis progression in DKD. Additionally, gut microbiota-regulated oxidative stress and inflammatory responses are now recognized as key factors in renal fibrogenesis, strengthening the gut–kidney axis in DKD progression (Li et al., 2024).

Mechanistically, this process is closely linked to innate immune regulation. Mitochondrial antiviral signaling protein (MAVS) plays a critical role in maintaining intestinal barrier function and preventing endotoxin leakage. When MAVS signaling is impaired, systemic inflammation and metabolic dysfunction worsen, accelerating DKD progression (Linh et al., 2022).

Gut microbiota dysbiosis profoundly impacts bile acid metabolism, acting as a critical link between metabolic and immune regulation. An imbalanced microbial community alters the expression of bile acid transporters and modifies the composition of the bile acid pool, disrupting normal signaling and homeostasis (Liu et al., 2022). Abnormal bile acid profiles—particularly an increased proportion of 12α-hydroxylated bile acids—can suppress the activity of the farnesoid X receptor (FXR) and the G protein-coupled bile acid receptor TGR5. Inhibition of these pathways exacerbates systemic and renal insulin resistance, promotes lipid accumulation, and creates a metabolic environment conducive to renal fibrosis (Zhou et al., 2016). These alterations reflect the interactions between gut-derived metabolites, oxidative stress, and renal inflammation (Tian et al., 2023; Corradi et al., 2024). Intercellular crosstalk forms a regulatory network within the local fibrotic microenvironment. Notably, the gut-kidney axis plays a key role in this network: levels of asymmetric dimethylarginine (ADMA) in urine and plasma are elevated in patients with DKD, and gut microbiota metabolism may influence ADMA levels (Schrauben et al., 2023). A prospective study of type 2 diabetes, involving both Asian and European cohorts, further supports the central role of the gut-kidney axis, showing that gut-derived methylamine metabolites are significantly associated with the progression to kidney failure. This establishes that gut microbiota-derived metabolic products can directly drive DKD deterioration (Saulnier et al., 2025). Furthermore, gut microbiota-derived uremic solutes are closely linked to cardiovascular mortality in patients with DKD, extending the pathological impact of the gut-kidney axis from renal fibrosis to systemic cardiovascular damage (Sapa et al., 2022). A systematic review and meta-analysis of type 2 diabetes metabolomes reinforced this connection, demonstrating shared blood metabolic features across DKD, cardiovascular disease (CVD), and diabetic retinopathy, positioning the gut-kidney axis as a critical mediator of multi-organ diabetic complications (Kuo et al., 2025).

Experimental studies demonstrate that activating FXR—using agonists such as GW4064—can mitigate high-glucose-induced damage. In human mesangial cells, FXR activation reduces inflammation, ECM deposition, and abnormal cell proliferation by downregulating the adipocytokine visfatin. In animal studies, FXR activation in db/db mice slows the progression of diabetic nephropathy. These findings highlight the antifibrotic and anti-inflammatory potential of the FXR signaling pathway (Zhou et al., 2016) (Table 1). Recent bibliometric analyses have identified the “gut–kidney axis,” short-chain fatty acids (SCFAs), probiotics, the intestinal barrier, inflammation, and renal fibrosis as core research areas in the field of gut microbiota and CKD, further supporting the pivotal role of gut dysbiosis in the progression of CKD and DKD (Tao et al., 2024).

Gut microbiota dysbiosis alters the expression of bile acid receptors and disrupts the balance of bile acid-derived metabolites (Xu J. et al., 2024). An abnormal bile acid profile promotes proinflammatory M1 macrophage polarization via the TLR2/NF-κB signaling pathway, leading to the release of cytokines such as IL-1β and TNF-α, which further intensify inflammation (Figure 2). Conversely, reduced levels of secondary bile acids, such as lithocholic acid derivatives 3-oxoLCA and isoalloLCA, disturb the balance between Tregs and Th17 cells. This imbalance favors Th17 cell expansion and increases the secretion of IL-17, IL-21, and IL-22. These changes foster a chronic inflammatory and profibrotic renal microenvironment (Xu J. et al., 2024). These immune–metabolic consequences align with recent findings demonstrating how gut-derived metabolites regulate Treg/Th17 differentiation and influence renal fibrosis (Shi et al., 2023; Stepanova, 2024).

In sum, the high glucose–gut microbiota–bile acid axis acts as a potent systemic amplifier and feedback regulator of metabolism–immune interactions in DKD. Hyperglycemia-induced gut dysbiosis, intestinal barrier disruption, endotoxemia, and bile acid signaling imbalance converge to exacerbate oxidative stress, chronic inflammation, and renal fibrosis. Targeting this axis—by restoring barrier integrity, normalizing bile acid composition, and selectively activating FXR/TGR5—may help break the vicious cycle of metabolic inflammation and fibrotic remodeling, offering a promising therapeutic strategy for DKD.

### 3.4 Mitochondrial damage: igniting the inflammatory drive to renal fibrosis

The fibrotic progression of DKD is heavily influenced by mitochondria, the primary site for energy metabolism and immune signaling. Chronic hyperglycemia impairs mitochondrial function by inhibiting OXPHOS and reducing the efficiency of the electron transport chain. This dysfunction disrupts cellular energy balance, leading to excessive ROS production and insufficient ATP generation (Forbes and Thorburn, 2018). Mitochondrial dynamics disorder and BNIP3-mediated mitophagy deficiency result in increased ROS production, which activates the ROS-P53-Bax/Bcl-2 pathway. This stabilization of P53 protein upregulates the pro-apoptotic protein Bax and downregulates the anti-apoptotic protein Bcl-2, thereby promoting tubular apoptosis and inflammation (Zhao et al., 2024). Through the release of DAMPs, this mitochondrial dysfunction not only reduces the energy supply in tubular epithelial cells but also triggers sterile inflammation (Aachoui et al., 2013). Notably, high glucose levels cause a significant upregulation of the novel DAMP high-mobility group nucleosome-binding protein 1 (HMGN1). HMGN1 activates the TLR4/MyD88/NF-κB signaling pathway, leading to the synthesis of inflammatory cytokines and macrophage infiltration, further exacerbating tubular damage and accelerating fibrosis (Yu et al., 2024) (Figure 2). Inhibition of HMGN1 significantly alleviates these pathological changes (Yu et al., 2024), demonstrating that DAMP release due to mitochondrial dysfunction is a critical link between fibrotic progression, innate immune activation, and metabolic stress. Potential anti-fibrotic interventions include inhibiting MFF/Drp1-mediated excessive mitochondrial fission, restoring mitophagy by interfering with the CERS6-ceramide-PINK1 axis, or upregulating circAASS expression to enhance mitochondrial quality control. However, the specific molecular interactions between these regulatory targets and their clinical applicability require further investigation (Song et al., 2024; Wang X. et al., 2023; Ma T. et al., 2025).

Infiltrating immune cells are another key site for metabolic–immune interactions, with functional polarization directly influenced by mitochondrial metabolism. Macrophages, in particular, are susceptible to these changes. In response to inflammatory stress and hyperglycemia, their metabolism shifts from OXPHOS to glycolysis. To meet the increased energy demand, this shift is accompanied by elevated expression of glucose transporters, including GLUT1 (Murphy and O’Neill, 2020; Thorens and Mueckler, 2010; Basso et al., 2021). This metabolic change dictates macrophage polarization, with glycolysis-dependent macrophages adopting a pro-inflammatory M1 phenotype, while OXPHOS- and fatty acid-oxidizing macrophages exhibit a profibrotic M2 phenotype (Darshi et al., 2024).

In addition to macrophages, other immune cells, particularly neutrophils, are also influenced by mitochondrial dysfunction in their pathogenic roles. Studies show that neutrophils upregulate ICAM-1 in response to endotoxin or high glucose stimulation, enhancing their phagocytic activity and increasing ROS production. Conversely, the loss of ICAM-1 weakens these effector functions, highlighting the pivotal role of mitochondrial ROS (mtROS) in neutrophil activation (Woodfin et al., 2016). Sterile inflammation is driven by the formation of neutrophil extracellular traps (NETs), which is facilitated by excessive mtROS (Gupta et al., 2022). Both patients with DKD and animal models exhibit significantly higher levels of NETs, which disrupt the glomerular endothelial barrier and activate the NLRP3 inflammasome. This leads to the release of IL-1β and IL-18, as well as increased collagen deposition (Shafqat et al., 2022). Pharmacological inhibition of NET formation, using agents such as PAD4 inhibitors or DNase I to degrade NETs, reduces endothelial dysfunction and kidney injury. These findings highlight the key role of NETs in the relationship between mitochondrial oxidative stress and renal fibrosis progression in DKD (Zheng et al., 2022).

Dysfunction of mitochondria–endoplasmic reticulum contact sites (MAMs) exacerbates metabolic stress and disrupts calcium homeostasis at the ultrastructural level. This dysfunction promotes fibrotic responses and increases NLRP3 inflammasome activation in macrophages. One critical link between metabolic stress and inflammation in DKD is MAM dysfunction. Under normal conditions, MAMs maintain cellular energy balance by regulating calcium (Ca^2+^) transfer between the endoplasmic reticulum and mitochondria. However, excessive MAM formation in a hyperglycemic environment leads to oxidative stress and mitochondrial Ca^2+^ overload. These changes accelerate disease progression by activating the NLRP3 inflammasome and triggering sterile inflammation (Wei et al., 2020). In podocytes, persistent NLRP3 activation accelerates glomerulosclerosis, disrupts autophagic balance, and stimulates caspase-1-mediated IL-1β release, significantly influencing DKD progression (Wei et al., 2020). On the other hand, excessive MAM formation is inhibited, and Ca^2+^ transfer from the endoplasmic reticulum to mitochondria is limited when the transient receptor potential channel TRPV1 is activated. This activation restores mitochondrial homeostasis via the AMPK–Fundc1 signaling pathway, mitigating renal fibrosis and podocyte damage (Shahzad et al., 2022). In summary, the MAM–Ca^2+^–NLRP3 axis may serve as a critical structural foundation for the metabolic–immune interaction that drives the onset and progression of DKD (Table 1).

### 3.5 Epigenetic reprogramming: memory mechanisms in metabolism–immunity crosstalk

Metabolic disruptions in DKD lead to persistent epigenetic reprogramming, establishing a “metabolic–inflammatory memory” that sustains fibrosis and alters immune cell activity. In this process, metabolic intermediates serve as key epigenetic modifiers. For example, excessive succinate in hyperglycemic conditions inhibits histone demethylases, maintaining hypermethylation at pro-inflammatory gene loci and locking macrophages into a persistent M1 phenotype (Tannahill et al., 2013). In contrast, α-ketoglutarate promotes tissue repair and inflammation resolution by facilitating DNA demethylation and encouraging macrophage polarization toward the reparative M2 phenotype (Tannahill et al., 2013). Additionally, reduced NAD^+^ levels impair SIRT1’s deacetylase activity, exacerbating inflammation and leading to continuous NF-κB activation (Wang W. et al., 2019).

Chronic hyperglycemia also increases the expression of DNA methyltransferase 1 (DNMT1), which silences negative regulators of the mTOR pathway through promoter methylation. In immune cells, this results in metabolic–inflammatory memory and chronic mTOR activation, advancing fibrosis, disrupting T-cell balance, and boosting cytokine production. The therapeutic potential of targeting this pathway is demonstrated by the use of DNMT inhibitors, such as 5-Aza, which help restore immune homeostasis (Chen et al., 2019) (Table 1).

This concept is further supported by recent findings. In DKD, the glycolytic enzyme PFKFB3 is upregulated, enhancing lactate production, which serves as a substrate for histone lactylation. This modification increases H4K12la marks at promoters of genes in the NF-κB pathway, such as Ikbkb, Rela, and Relb, directly triggering their transcription and maintaining immune cell infiltration and inflammatory signaling (Wang Y. et al., 2024). Macrophages also undergo similar lactylation, with H3K18la modification promoting the long-term expression of inflammatory genes like TNF-α and IL-6. This mechanism contributes to DKD progression by perpetuating chronic inflammation (Shi et al., 2025; Chu et al., 2021). These results suggest that lactate-driven epigenetic remodeling establishes long-term “immune memory” in various cell types, sustaining fibrosis and inflammation even after the initial metabolic stress has subsided (Figure 2).

Moreover, N6-methyladenosine (m6A) modification has emerged as an important regulatory mechanism in immune metabolism and inflammation (Wang H. et al., 2025), although research in DKD is still limited. Current evidence indicates that METTL3-dependent m6A methylation promotes renal fibrosis (Jung et al., 2024). Bioinformatic analyses have identified m6A-related biomarkers, such as CTSS and CD53, which are closely associated with immune cell infiltration in DKD (Leilei et al., 2025). However, the direct link between m6A modification, macrophage polarization, and metabolic regulation remains unclear. Recent studies (Chen et al., 2024; Chen and Natarajan, 2022) suggest that ATP-dependent chromatin remodeling affects chromatin accessibility, maintaining the activation of profibrotic genes under chronic hyperglycemia or metabolic stress. This sustained “open chromatin” state creates a memory-like vulnerability to fibrosis, pointing to a promising direction for future research on the epigenetic mechanisms underlying metabolism–immune crosstalk in DKD (Table 1).

## 4 Therapeutic strategies targeting immuno-metabolic crosstalk

### 4.1 Metabolic-targeted therapies

Mitochondrial dysfunction is a key contributor to renal fibrosis in DKD, prompting increasing attention to therapeutic strategies aimed at restoring energy metabolism. Mitochondrial protectants, such as MitoQ, CoQ10, and SS-31, reduce mtROS production and restore membrane potential, thereby mitigating tubular and endothelial cell damage and supporting mitochondrial and cellular recovery (Ward et al., 2017; Xiao et al., 2017). Additionally, substances that regulate energy metabolism offer promising therapeutic potential. For example, the AMPK activator metformin enhances fatty acid oxidation while inhibiting glycolysis, helping to balance cellular energy levels and reduce oxidative stress (Sun et al., 2021). SIRT1 activators, such as resveratrol, promote the activity of PGC-1α by deacetylating it, which not only stimulates mitochondrial biogenesis and functional restoration but also impacts lipid metabolism and reduces inflammatory signaling (Yacoub et al., 2014). HIF stabilizers, such as enarodustat and FG-4592, are also noteworthy. These compounds enhance kidney energy metabolism, reduce oxidative stress, and inhibit macrophage infiltration and cytokine production, helping to alleviate early-stage kidney damage and potentially preventing the activation of pro-fibrotic signaling pathways (Bessho et al., 2019; Li et al., 2020; Hasegawa et al., 2020).

Metabolic interventions not only target the kidneys but also exert systemic effects through the gut–kidney axis. Studies have shown that certain substances, such as metformin, SGLT2 inhibitors, pirfenidone, and various natural polysaccharides, can remodel the gut microbiota, altering its metabolic activity in beneficial ways. These changes help restore the balance between immune and metabolic functions, reduce inflammation, and protect the kidneys from damage (Guo et al., 2025; Mao et al., 2023). Small-molecule modulators, such as lactate dehydrogenase (LDH) inhibitors and SUCNR1 antagonists, directly influence immune cell metabolism. This can enhance renal tubule function, reduce local inflammation, and help restore metabolic balance (Wang Z. et al., 2019; Zhang et al., 2024). While current research is still in its early stages, these strategies show significant promise, highlighting new potential targets for developing metabolic–immune interventions in DKD (Table 2).

**TABLE 2:** Therapeutic strategies targeting metabolic–immune crosstalk in diabetic kidney disease (DKD).

Category	Main strategies/Drugs	Mechanism of action	Refs
Metabolic-targeted therapies	Mitochondrial protectants (MitoQ, CoQ10, SS-31)	Lower mitochondrial ROS and restore membrane potential to protect tubular and endothelial cells	Xiao et al. (2017) Sun et al. (2021)
	AMPK activators (Metformin)	Enhance FAO and suppress glycolysis to improve cellular energy balance and reduce oxidative stress	Yacoub et al. (2014)
	SIRT1 activators (Resveratrol, etc.)	Activate PGC-1α via deacetylation to promote mitochondrial biogenesis and suppress metabolic inflammation	Bessho et al. (2019)
	HIF stabilizers (Enarodustat, FG-4592)	Improve renal energy metabolism, reduce oxidative stress, and limit macrophage infiltration and cytokine production	Li et al. (2020) Hasegawa et al. (2020) Guo et al. (2025)
	Gut–kidney axis modulators (Metformin, SGLT2 inhibitors, Pirfenidone, polysaccharide-based natural compounds)	Remodel gut microbiota to restore immunometabolic balance and reduce systemic and renal inflammation	Mao et al. (2023) Wang Z. et al. (2019)
	Small-molecule metabolic modulators (LDH inhibitors, SUCNR1 antagonists)	Modulate immune-cell metabolism, reduce inflammatory activation, and improve tubular function	Zhang et al. (2024) O’Brien et al. (2017)
Immune-targeted therapies	CCR2/CCR5 antagonists (MK-0812, PF-04634817)	Reduce monocyte/macrophage recruitment and attenuate inflammasome-mediated inflammation	Wang J. et al. (2024) Gale et al. (2018)
	Stem cell therapy (placental MSCs)	Restore Th17/Treg balance through PD-1/PD-L1 signaling and reduce inflammatory cytokine production	Qu et al. (2025)
	Epigenetic intervention (DNA demethylation agent 5-Aza)	Reverse DNMT-dependent hypermethylation to suppress Wnt signaling and reduce ECM accumulation	Shaman et al. (2022)
Integrated immuno-metabolic therapies	GLP-1 receptor agonists (Semaglutide)	Improve glucose–lipid metabolism, suppress TNF-α/IL-1β, and enhance macrophage phagocytosis	Jastreboff et al. (2022) Pratley et al. (2024) Naaman and Bakris (2023) Shao et al. (2022) Cao et al. (2025)
	SGLT2 inhibitors (Empagliflozin)	Enhance FAO and inhibit NLRP3/TLR4–NF-κB activation to reduce renal inflammation	Rykova et al. (2025) Yang et al. (2022) Wu et al. (2025) Zhang et al. (2025)
	Metabolic modulator DMM (Dimethyl malonate)	Inhibit SDH to promote PPAR-mediated FAO, reduce mtROS/CD4^+^ infiltration, and attenuate fibrosis	Vergadi et al. (2017)
	mTOR/PI3K–Akt pathway modulators (Formononetin)	Regulate immune-cell metabolic programming and inhibit TGF-β/Smad-driven fibrotic signaling	Chen L. et al. (2023) Song et al. (2025) Xu J. et al. (2024) Jiang et al. (2007)
	FXR agonists	Improve bile acid metabolism and suppress NF-κB–mediated macrophage inflammation and fibrosis	Sun et al. (2023), Kim et al. (2023), Liang D. et al. (2024)
	FGF21 analogs	Promote FAO, improve mitochondrial homeostasis, and inhibit NLRP3-mediated inflammatory signaling	Weng et al. (2020) Zhao et al. (2017) Chang et al. (2021) Xu et al. (2025)
Traditional Chinese Medicine (TCM) and natural compounds	Astragalus polysaccharides (APS)	Activate AMPK/PGC-1α to enhance mitochondrial metabolism, insulin sensitivity, and gut microbiota balance	Guo et al. (2023) Sui et al. (2023)
	Ginsenoside Rg3	Activate SIRT1/AMPK, improve metabolic homeostasis, and suppress TGF-β/Smad fibrosis	Lu et al. (2018)
	Quercetin	Reduce oxidative stress and inflammation via SIRT1/PI3K/Akt/mTORC1 regulation and alleviate fibrosis	Liu et al. (2019) Ma et al. (2024)
	Resveratrol	Modify epigenetic metabolic memory via SIRT1/3 activation or HDAC inhibition to reduce fibrosis	Xu et al. (2025)
	TCM mixtures	Remodel gut microbiota, enhance SCFA production, and improve gut–kidney immune-metabolic tolerance	Sui et al. (2023) Shen et al. (2023) Wang Y. et al. (2025)

### 4.2 Immune-targeted therapies

Interventions targeting immune signaling pathways primarily aim to reduce inflammation, restore immune system balance, and regulate immune cell activity. Among these pathways, the C-C chemokine receptors CCR2 and CCR5 play a critical role in recruiting monocytes and macrophages. Over-activation of these receptors is closely linked to diabetes-related inflammation. Dual CCR2/CCR5 antagonists, such as MK-0812 and PF-04634817, effectively reduce the infiltration of pro-inflammatory M1 macrophages and promote the polarization of M2 macrophages, alleviating tissue inflammation and improving insulin resistance (O’Brien et al., 2017). In a phase II clinical trial involving patients with DKD, PF-04634817, when combined with standard therapy (ACEI/ARB), significantly reduced albuminuria levels. Although the overall effect was moderate, the treatment was deemed safe. These findings highlight the CCR2/CCR5 axis as a promising target for immune modulation in DKD (Gale et al., 2018).

In addition to pharmacological interventions, cell-based therapies offer significant potential for restoring immune homeostasis. Human placental MSCs (PMSCs) activate the programmed death 1/programmed death ligand 1 (PD-1/PD-L1) signaling pathway, rebalance Th17 and Treg populations, and suppress pro-inflammatory cytokines such as IL-17A and IL-1β. These actions significantly improve renal function and reduce histopathological damage in DKD rat models (Wang J. et al., 2024).

Moreover, epigenetic modulation presents a novel therapeutic approach for immune regulation. In DKD, overexpression of DNA methyltransferase DNMT3B promotes ECM accumulation and renal fibrosis by methylating and silencing SFRP5, an inhibitor of the Wnt pathway. This silencing activates the Wnt/β-catenin signaling cascade, driving fibrosis progression. Treatment with the demethylating agent 5-Aza reverses these epigenetic changes, reduces ECM deposition and inflammation, and slows DKD progression (Qu et al., 2025) (Table 2).

Overall, immune-targeted therapies not only mitigate tissue injury by inhibiting inflammasome activation and pro-inflammatory cytokine release but also reshape the immune microenvironment to restore metabolic balance. These dual effects provide both a theoretical framework and a practical foundation for developing integrated immuno-metabolic interventions in DKD.

### 4.3 Integrated immuno-metabolic therapies

Combination therapies targeting both metabolic and immune pathways represent a promising new approach for treating DKD. Glucagon-like peptide-1 receptor agonists (GLP-1RAs), such as semaglutide and liraglutide, have shown significant renal protective effects beyond glucose lowering in several large cardiovascular outcome trials, including SUSTAIN-6, LEADER, and AMPLITUDE-O. These agents reduced urinary albumin excretion by approximately 24%, slowed the annual decline in estimated glomerular filtration rate (eGFR) by 0.3–0.9 mL/min/1.73 m^2^, and provided greater benefits in patients with baseline eGFR values below 60 mL/min/1.73 m^2^ (Shaman et al., 2022; Jastreboff et al., 2022; Pratley et al., 2024). Mechanistically, GLP-1RAs enhance glucose and lipid metabolism while suppressing the release of pro-inflammatory cytokines such as TNF-α and IL-1β. They also promote M2 macrophage polarization and reduce MCP-1 expression, mitigating renal inflammation and fibrosis. Additionally, GLP-1RAs significantly improve body weight control and insulin sensitivity, which helps alleviate obesity-associated chronic inflammation (Naaman and Bakris, 2023; Shao et al., 2022).

Similarly, sodium-glucose cotransporter-2 (SGLT2) inhibitors, such as empagliflozin and dapagliflozin, enhance energy metabolism and promote fatty acid oxidation. Concurrently, they suppress activation of the NLRP3 inflammasome and the TLR4/NF-κB signaling pathway, thereby reducing inflammation and protecting renal function (Cao et al., 2025; Rykova et al., 2025). These agents inhibit M1 macrophage polarization and help restore an anti-inflammatory metabolic phenotype. When combined with renin–angiotensin system (RAS) inhibitors or mineralocorticoid receptor antagonists (MRAs), SGLT2 inhibitors act synergistically to reduce proteinuria, lower the risk of hyperkalemia, and improve long-term renal outcomes (Yang et al., 2022; Wu et al., 2025) (Table 2).

Furthermore, Zhang et al. (Zhang et al., 2025) demonstrated in an obstructive nephropathy model that dimethyl malonate (DMM) alleviates renal inflammation and fibrosis by inhibiting mitochondrial succinate dehydrogenase (SDH). This inhibition promotes metabolic reprogramming and activates the peroxisome proliferator-activated receptor (PPAR) pathway, enhancing fatty acid oxidation and improving mitochondrial function. DMM also reduces mtROS production and CD4^+^ T-cell infiltration, thereby suppressing NF-κB–mediated cytokine release and interstitial inflammation. Although these findings were derived from an obstructive nephropathy model rather than DKD, the underlying mechanism—targeting mitochondrial metabolism to restore energy balance and indirectly regulate immune inflammation—suggests that DMM may be a promising therapeutic candidate for metabolic renal fibrosis, warranting further investigation in DKD.

Building on this concept, formononetin—a modulator of the mTOR/PI3K–Akt pathway—emerges as a dual regulator of metabolism and immunity. It influences metabolic programming in immune cells (Vergadi et al., 2017), promotes anti-inflammatory polarization, and suppresses TGF-β/Smad–mediated fibrotic signaling. Through these actions, formononetin effectively alleviates renal interstitial fibrosis in DKD (Chen L. et al., 2023; Song et al., 2025; Xu F. et al., 2024) (Table 2).

Moreover, activation of FXR reduces the expression of MCP-1 and visfatin, limits macrophage cytokine release, and inhibits NF-κB activation. These combined effects help mitigate mesangial expansion and tubulointerstitial fibrosis (Jiang et al., 2007; Sun et al., 2023; Kim et al., 2023).

FGF21 analogs also play a role in restoring immunometabolic balance. These compounds enhance fatty acid oxidation, improve mitochondrial dynamics, and inhibit NLRP3 inflammasome activity (Liang Y. et al., 2024; Weng et al., 2020; Zhao et al., 2017). As a result, levels of IL-1β and TGF-β1 decrease, and ECM accumulation is reduced. A prospective clinical study involving 312 patients revealed that elevated serum FGF21 levels were significantly associated with a >30% decline in eGFR and worsening albuminuria. Furthermore, combining FGF21 with soluble TNF receptor 1 (sTNFR1) improved the prediction accuracy of renal function deterioration (adjusted HR 4.45, 95% CI 1.86–10.65, *p* = 0.001). These findings suggest that FGF21 acts not only as a mechanistic regulator of immunometabolic crosstalk but also as a predictive biomarker linking metabolic reprogramming to renal outcomes in DKD (Chang et al., 2021).

These emerging drugs and biomarkers target the interconnected network between metabolism and immunity, forming a comprehensive treatment framework that integrates energy metabolism remodeling, inflammation inhibition, and fibrosis reversal.

### 4.4 Traditional Chinese Medicine and natural compounds

Traditional Chinese Medicine (TCM) and natural products exhibit integrative multi-target characteristics in DKD. Astragalus polysaccharide (APS) activates the AMPK/PGC-1α axis to improve insulin sensitivity and mitochondrial energy metabolism, enhance lipid oxidation, and suppress oxidative stress (Xu et al., 2025; Guo et al., 2023). Ginsenoside Rg3 activates the SIRT1/AMPK pathway to alleviate insulin resistance, promote metabolic balance, upregulate the anti-inflammatory cytokine IL-10, and inhibit TGF-β/Smad signaling, achieving bidirectional regulation of metabolism and immunity (Sui et al., 2023). Quercetin exerts anti-inflammatory, antioxidant, and antifibrotic effects by modulating SIRT1, PI3K/Akt, and mTORC1 pathways, alleviating ER stress and cellular senescence, and reducing M1 macrophage infiltration (Lu et al., 2018; Liu et al., 2019) (Table 2).

TCM also affects epigenetic regulation and microecological remodeling. Various formulas and monomers modulate genome-wide or locus-specific DNA methylation via DNMT1/3, influencing oxidative stress, inflammation, mitochondrial dysfunction, and lipid metabolism in DKD (Ma et al., 2024). Resveratrol further alters the epigenetic state of metabolism-related genes through SIRT1/3 activation or HDAC inhibition, contributing to an antifibrotic “metabolic memory” (Huang et al., 2014). In parallel, polysaccharides and herbal prescriptions—such as Astragalus–Salvia miltiorrhiza combinations and Shen-ling-bai-zhu-san—reshape gut microbiota composition, enhance SCFA production, and improve gut–kidney immune-metabolic tolerance (Xu et al., 2025; Guo et al., 2023; Shen et al., 2023). These effects are particularly relevant due to the role of gut-derived uremic toxins and AHR ligands in driving inflammation and fibrosis.

Recent studies highlight a convergent mechanism in which TCM monomers regulate the AHR–NF-κB–Nrf2 axis, a key immunometabolic pathway linking ROS overload, toxin accumulation, inflammation, and fibrotic remodeling. Acteoside acts as an AHR antagonist, inhibiting AHR nuclear translocation, suppressing NF-κB activation, enhancing Nrf2-mediated antioxidative responses, and alleviating adenine-induced fibrosis (Wang Y. et al., 2025). Geniposidic acid similarly represses AHR signaling, downregulates NF-κB target genes, and activates Nrf2-dependent antioxidative pathways, mitigating chronic tubulointerstitial nephropathy (Wang Y-N. et al., 2024).

Notably, cross-disciplinary evidence suggests that certain TCM compounds also modulate microvascular and hypoxia-related pathways. Curcumin, tanshinone IIA, and ginsenosides influence key angiogenic signals, including HIF-1α and VEGF/VEGFR (Wang W-Y. et al., 2025). Given that DKD is characterized by PTC rarefaction and persistent renal hypoxia, these mechanisms provide indirect support for the microvascular protective potential of TCM in DKD.

In summary, TCM and its active constituents integrate metabolic regulation, immune suppression, epigenetic reprogramming, microecological remodeling, redox balance, and microvascular protection to counteract DKD-related renal fibrosis. These multimodal actions provide a mechanistic and pharmacological foundation for developing combinatorial immunometabolic therapies and enhancing synergy with modern agents such as SGLT2 inhibitors and GLP-1 receptor agonists.

## 5 Conclusion and perspectives

DKD-related renal fibrosis results from the cumulative effects of metabolic stress, immune activation, and microvascular damage, creating a self-reinforcing immuno-metabolic loop that accelerates disease progression. Growing evidence indicates that disruptions in glucose and lipid metabolism, mitochondrial dysfunction, and gut-derived metabolites alter immune cell behavior, amplifying inflammatory and profibrotic signaling. This highlights immuno-metabolism as a critical therapeutic target. Current treatments—including SGLT2 inhibitors and GLP-1 receptor agonists, which improve metabolic profiles while suppressing NLRP3 inflammasome activation and enhancing regulatory T-cell function (Joumaa et al., 2025)—as well as multi-component traditional Chinese medicines that regulate energy metabolism, inflammation, epigenetic states, and gut–kidney homeostasis (Li et al., 2025; Cai et al., 2025), demonstrate the promise of dual-target regulation. Emerging strategies, such as nanodelivery systems (He et al., 2022) and combination therapies (Liang D. et al., 2024), further expand the potential for precisely targeting immuno-metabolic pathways. However, many studies remain associative rather than causal, and most mechanistic insights are based on rodent models with limited translational relevance. This review may also have selective omissions due to scope constraints. Nonetheless, converging evidence highlights immuno-metabolic crosstalk as a central driver of DKD fibrosis. Moving forward, integrating human-tissue multi-omics, immune–metabolic profiling, and targeted therapeutic strategies will be critical for developing precision interventions to disrupt this pathogenic network.
