# Supplementary material for: Immune-metabolic interactions shape the fibrotic landscape of diabetic kidney disease: emerging mechanisms and therapeutic prospects
Source: Front Physiol. 2026 Jan 21;16:1736472. doi: 10.3389/fphys.2025.1736472 (PMC12867825; doi:10.3389/fphys.2025.1736472)
Supplement: Supplementary file 1 [file DataSheet1.docx]

Supplementary Material

# Supplementary Figures and Tables

## Supplementary Figures


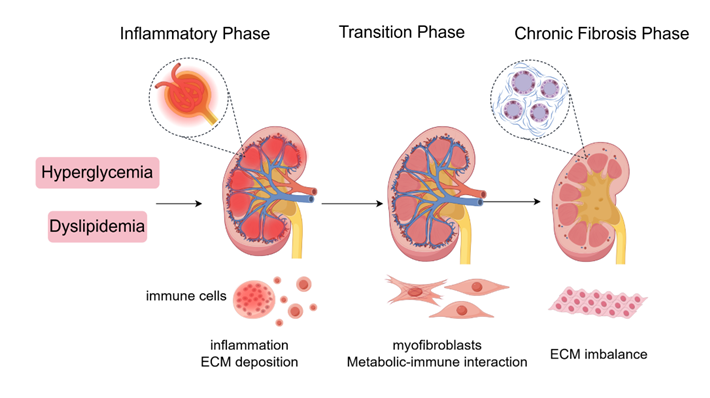


## Figure 1. Stages of diabetic kidney disease (DKD) fibrosis. Persistent hyperglycemia and dyslipidemia drive DKD progression from inflammation to fibrosis. The inflammatory phase features immune activation and ECM deposition; the transition phase involves metabolic–immune interaction and myofibroblast activation; and the chronic fibrosis phase is characterized by ECM imbalance and irreversible renal scarring.


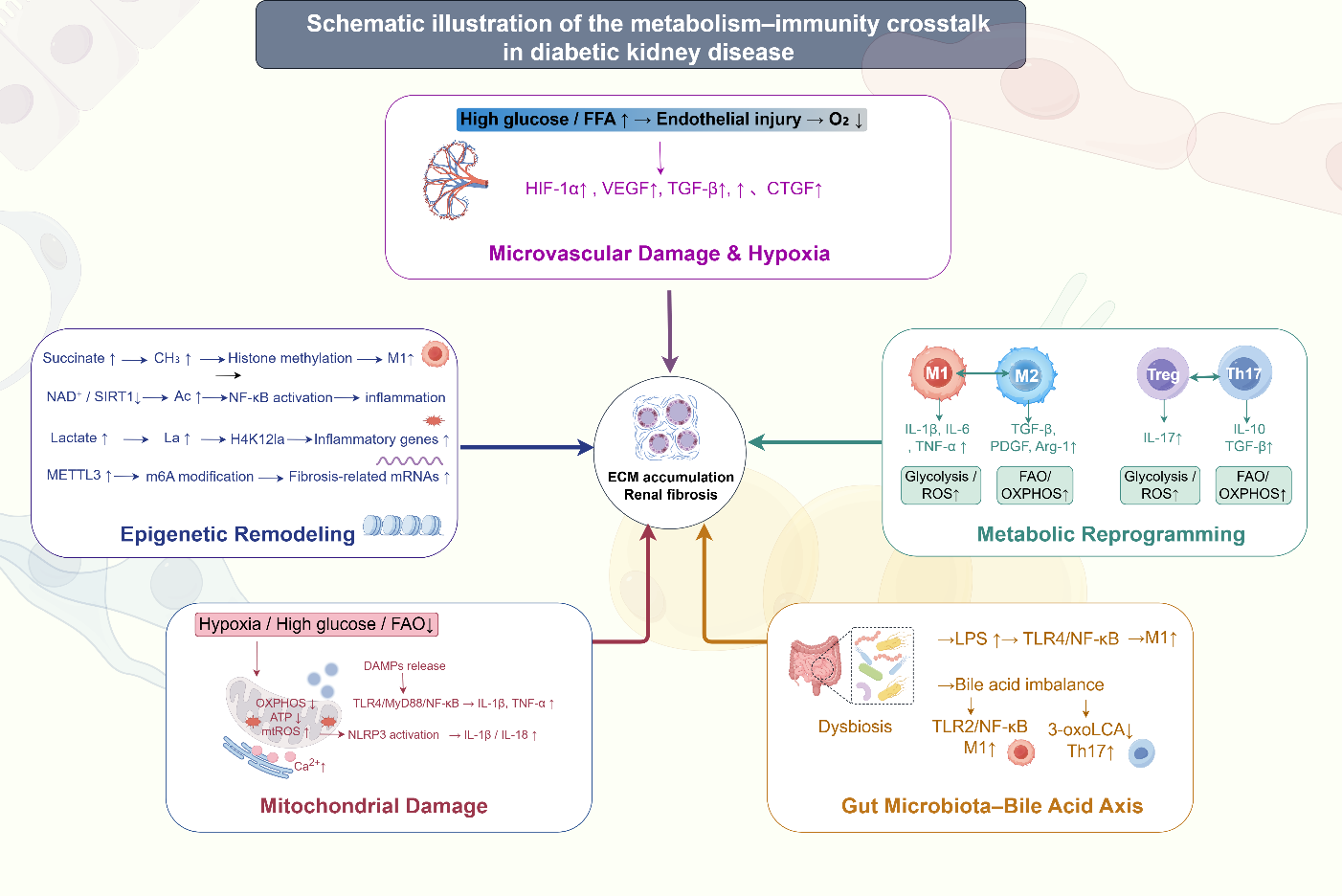


**Figure 2. Metabolism–immunity crosstalk in diabetic kidney disease (DKD).** Chronic metabolic stress in diabetes triggers microvascular injury, hypoxia, and metabolic reprogramming in renal and immune cells. These changes enhance immune cell infiltration and polarization (M1/M2 macrophages, Th17/Treg cells), while gut dysbiosis and bile acid imbalance amplify systemic inflammation. Mitochondrial dysfunction releases ROS and DAMPs, activating innate immune pathways and promoting fibrosis. Metabolic intermediates such as succinate, NAD⁺, and lactate further drive epigenetic remodeling, forming a persistent inflammatory and fibrotic state.

## Supplementary Tables

## Table 1. Mechanistic summary of metabolism–immunity crosstalk driving renal fibrosis in diabetic kidney disease (DKD)

| Interaction | Key Pathways / Molecules | Mechanism | Impact on DKD Fibrosis |
| --- | --- | --- | --- |
| High glucose–metabolite–immune activation | Glucose, FFA, BCAA/BCKA, lactate, succinate | Metabolic intermediates directly induce pro-inflammatory phenotypes of immune cells (M1/M2 macrophages, Th17), while cytokines suppress mitochondrial OXPHOS in tubular/endothelial cells, shifting metabolism toward glycolysis | Forms a positive feedback loop that accelerates ECM deposition and renal fibrosis |
| Microvascular injury and hypoxia | GBM thickening, PTC rarefaction, HIF-1α, TGF-β1, VEGF, CTGF | Capillary rarefaction causes local hypoxia; chronic HIF-1α activation upregulates fibrogenic signals | Initiates fibroblast activation and ECM accumulation, establishing fibrotic foundation |
| Immune cell metabolic reprogramming | M1/M2 macrophage glycolysis/OXPHOS; Th17 glycolysis; Treg fatty acid oxidation | M1 macrophages rely on glycolysis to release cytokines; M2 depend on OXPHOS and FAO but secrete TGF-β and PDGF; T-cell metabolic imbalance disrupts immune homeostasis | Amplifies local inflammation, promotes tubular injury and fibrosis |
| High glucose–gut microbiota–bile acid axis | LPS–TLR4, TLR2/NF-κB, FXR/TGR5, 3-oxoLCA | Gut dysbiosis produces metabolic endotoxemia and systemic inflammation; altered bile acid profile disrupts Treg/Th17 balance | Systemic inflammation and local metabolic disorder reinforce each other, promoting fibrosis |
| Mitochondrial damage | Impaired OXPHOS, ROS, DAMPs, HMGN1, MAMs, NLRP3 inflammasome | High glucose damages mitochondrial function, releasing DAMPs that activate innate immunity; macrophage glycolysis/FAO status determines polarization | Amplifies inflammation, strengthens fibrogenic signaling, exacerbates tubular and interstitial injury |
| Epigenetic remodeling | SIRT1/3, DNMT1, m⁶A, histone lactylation (H4K12la, H3K18la), PFKFB3 | Metabolic intermediates (succinate, α-KG, lactate) regulate DNA/histone modifications, maintaining pro-inflammatory/pro-fibrotic immune phenotypes | Establishes long-term “immune memory,” perpetuating metabolic–inflammatory imbalance and chronic fibrosis |
| Immuno-metabolic signaling crosstalk | NF-κB, JAK/STAT, PI3K/Akt, AMPK, mTOR | Chronic inflammation disrupts metabolic homeostasis, while metabolic stress enhances immune activation | Maintains a vicious cycle, accelerating DKD progression and renal function decline |

## Table 2. Therapeutic strategies targeting metabolic–immune crosstalk in diabetic kidney disease (DKD)

| Category | Main Strategies / Drugs | Mechanism of Action |
| --- | --- | --- |
| Metabolic-targeted therapies | Mitochondrial protectants (MitoQ, CoQ10, SS-31) | Reduce ROS generation, restore mitochondrial membrane potential, and alleviate tubular and endothelial injury |
|  | AMPK activators (Metformin) | Enhance fatty acid oxidation, inhibit glycolysis, improve energy homeostasis, and reduce oxidative stress |
|  | SIRT1 activators (Resveratrol, etc.) | Deacetylate and activate PGC-1α, promote mitochondrial biogenesis and function recovery, regulate lipid metabolism and inflammation |
|  | HIF stabilizers (Enarodustat, FG-4592) | Improve renal energy metabolism, reduce oxidative stress, and suppress macrophage infiltration and cytokine production |
|  | Gut–kidney axis modulators (Metformin, SGLT2 inhibitors, Pirfenidone, polysaccharide-based natural compounds) | Regulate gut microbiota composition and metabolic activity, restore immune–metabolic balance, and reduce renal inflammation and injury |
|  | Small-molecule metabolic modulators (LDH inhibitors, SUCNR1 antagonists) | Interfere with immune cell metabolic reprogramming, improve tubular function, and attenuate local inflammation |
| Immune-targeted therapies | CCR2/CCR5 antagonists (MK-0812, PF-04634817) | Reduce monocyte/macrophage recruitment and inflammasome activation |
|  | Stem cell therapy (human placental mesenchymal stem cells) | Modulate PD-1/PD-L1 signaling, restore Th17/Treg balance, and improve renal function and histopathology |
|  | Epigenetic intervention (DNA demethylation agent 5-Aza) | Regulate Wnt and E2F1 signaling pathways, slowing DKD progression |
| Integrated immuno-metabolic therapies | GLP-1 receptor agonists (Semaglutide) | Improve glucose–lipid metabolism, suppress TNF-α/IL-1β, and enhance macrophage phagocytosis |
|  | SGLT2 inhibitors (Empagliflozin) | Inhibit NLRP3 inflammasome activation under high-glucose conditions, improve renal energy metabolism, and modulate immune microenvironment |
|  | Metabolic modulator DMM (Dimethyl malonate) | Inhibits mitochondrial succinate dehydrogenase (SDH), promotes PPAR-mediated FAO and energy homeostasis, reduces mtROS and CD4⁺ T-cell infiltration, and mitigates renal fibrosis; a potential therapeutic candidate for metabolic renal fibrosis |
|  | mTOR/PI3K–Akt pathway modulators (Formononetin) | Regulate immune cell metabolic selection and inhibit TGF-β/Smad fibrotic signaling |
|  | FXR agonists | Improve bile acid metabolism, suppress NF-κB signaling and macrophage inflammation |
|  | FGF21 analogs | Promote fatty acid oxidation and suppress NLRP3 inflammasome activity |
| Traditional Chinese Medicine (TCM) and natural compounds | Astragalus polysaccharides (APS) | Activate AMPK/PGC-1α signaling, improve insulin sensitivity and mitochondrial energy metabolism, regulate gut microbiota |
|  | Ginsenoside Rg3 | Activate SIRT1/AMPK axis, improve glucose–lipid metabolism, suppress TGF-β/Smad-mediated fibrosis, and modulate immunity |
|  | Quercetin | Exert anti-inflammatory and antioxidant effects, regulate SIRT1/PI3K/Akt/mTORC1 signaling, and alleviate fibrosis and ER stress |
|  | Resveratrol | Activate SIRT1/3 or inhibit HDACs, modulate epigenetic states of metabolism-related genes, and establish anti-fibrotic “metabolic memory” |
|  | TCM and natural compound mixtures | Remodel gut microbiota, promote SCFA production, and enhance gut–kidney axis metabolism and immune tolerance |
